# New Structural Variants of Aeruginosin Produced by the Toxic Bloom Forming Cyanobacterium *Nodularia spumigena*

**DOI:** 10.1371/journal.pone.0073618

**Published:** 2013-09-06

**Authors:** David P. Fewer, Jouni Jokela, Eeva Paukku, Julia Österholm, Matti Wahlsten, Perttu Permi, Olli Aitio, Leo Rouhiainen, Gonzalo V. Gomez-Saez, Kaarina Sivonen

**Affiliations:** 1 Department of Food and Environmental Sciences, Division of Microbiology and Biotechnology, University of Helsinki, Helsinki, Finland; 2 Program in Structural Biology and Biophysics, Institute of Biotechnology, University of Helsinki, Helsinki, Finland; University of New South Wales, Australia

## Abstract

*Nodularia spumigena* is a filamentous diazotrophic cyanobacterium that forms blooms in brackish water bodies. This cyanobacterium produces linear and cyclic peptide protease inhibitors which are thought to be part of a chemical defense against grazers. Here we show that *N. spumigena* produces structurally novel members of the aeruginosin family of serine protease inhibitors. Extensive chemical analyses including NMR demonstrated that the aeruginosins are comprised of an N-terminal short fatty acid chain, L-Tyr, L-Choi and L-argininal and in some cases pentose sugar. The genome of *N. spumigena* CCY9414 contains a compact 18-kb aeruginosin gene cluster encoding a peptide synthetase with a reductive release mechanism which offloads the aeruginosins as reactive peptide aldehydes. Analysis of the aeruginosin and spumigin gene clusters revealed two different strategies for the incorporation of N-terminal protecting carboxylic acids. These results demonstrate that strains of *N. spumigena* produce aeruginosins and spumigins, two families of structurally similar linear peptide aldehydes using separate peptide synthetases. The aeruginosins were chemically diverse and we found 11 structural variants in 16 strains from the Baltic Sea and Australia. Our findings broaden the known structural diversity of the aeruginosin peptide family to include peptides with rare N-terminal short chain (C_2_–C_10_) fatty acid moieties.

## Introduction

*N. spumigena* is a filamentous diazotrophic cyanobacterium that forms extensive summer blooms in brackish water bodies. The ability to fix atmospheric nitrogen confers a competitive advantage on *N. spumigena* in nitrogen-poor and iron-limited brackish water ecosystems [1], [2], [3]. *N. spumigena* is responsible for a large part of the new nitrogen input in the Baltic Sea and is a source of environmental concern [1]. The consumption of water containing *N. spumigena* is associated with the death of wild and domestic animals [4], [5], [6]. These blooms are toxic through the production of nodularin, a cyclic pentapeptide toxin [4], [5]. Nodularin is the end-product of a complex hybrid non-ribosomal peptide synthetase (NRPS) and polyketide synthase (PKS) biosynthetic pathway [5]. *N. spumigena* produces other non-ribosomal peptides in addition to nodularin, including spumigin and nodulapeptin [7], [8], [9].

Spumigins are linear peptides which contain an N-terminal hydroxyphenyl lactic acid, almost exclusively D-homotyrosine, proline or 4*-*methylproline (mPro), and a C-terminal lysine or arginine derivative [7], [8], [9]. Spumigins are assembled on a NRPS enzyme complex which offloads the peptides as reactive aldehydes [8]. The enzymatic steps necessary for the synthesis of the unusual mPro are encoded together with the peptide synthetases in the 21-kb spumigin gene cluster [8]. Spumigins are potent inhibitors of serine proteases, active in the micromolar to nanomolar range [7], [8], [10].

Aeruginosins are another family of serine protease inhibitors that have been described from aquatic bloom-forming genera of cyanobacteria [11]. This family of linear peptides contain the rare 2-carboxy-6-hydroxyoctahydroindole (Choi) moiety as well as the C-terminal arginine derivatives argininal, argininol, agmatine, 1-amidino-2-ethoxy-3-aminopiperidine and more rarely 1-amino-2-(*N*-amidino-Δ^3^-pyrrolinyl)-ethyl moiety [11]. The N-terminus typically consists of either hydroxyphenyl lactic acid in *Microcystis* or phenyl lactic acid in *Planktothrix* [11]. Aeruginosins are the end-products of highly variable NRPS biosynthetic pathways and may be modified to contain chlorine, sulfate or sugars [12], [13]. Chemical variation in the aeruginosin structure is achieved by the action of tailoring enzymes and variation in the loading and release mechanisms [12], [13], [14]. A close relationship between aeruginosins and spumigins has been suspected for some time [12], [13], [15], [16].

**Figure 1:**
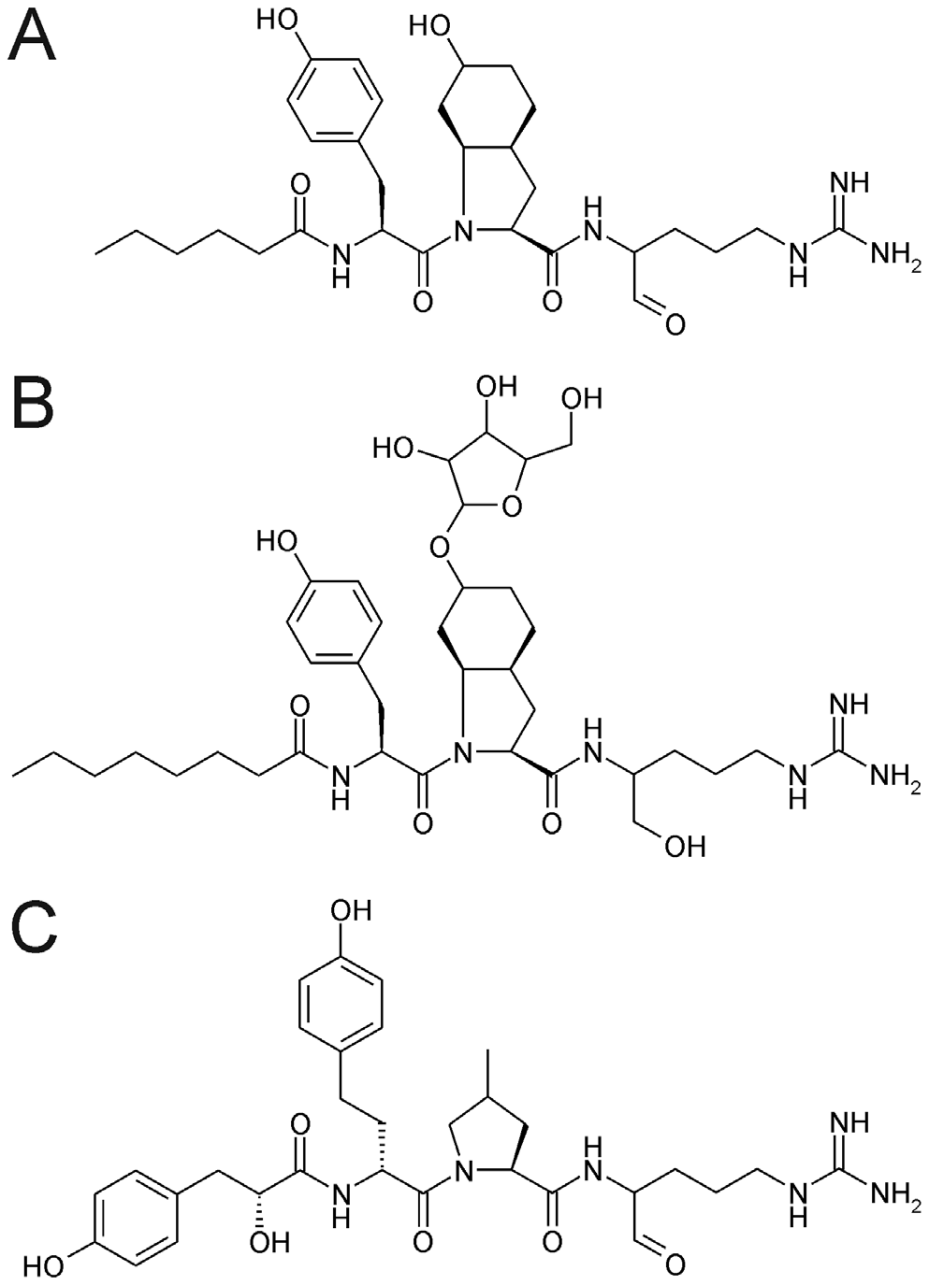
Representatives of the aeruginosin and spumigin families. These tetrapeptides are produced by strains of *Nodularia spumigena* isolated from brackish water bodies in Australia and the Baltic Sea. (A) aeruginosin NAL2, (B) an aeruginosin NOL6 containing *O*-linked pentose, (C) spumigin E.

*N. spumigena* encodes a number of cryptic NRPS clusters for which end-products have not been characterized [3], [17]. It was proposed based on the presence of Choi biosynthetic genes that *N. spumigena* may produce aeruginosins [3]. A recent study reported an incomplete peptide structure which contains Choi from *N. spumigena* [18]. Here we show that *N. spumigena* produces new members of the aeruginosin family of protease inhibitors using extensive chemical analyses including NMR studies (Figure 1) and demonstrate that *N. spumigena* strains produce two classes of similar non-ribosomal peptides, aeruginosins and spumigins, simultaneously using separate peptide synthetases. These results broaden the structural diversity of the aeruginosin family of peptides to include peptides with fatty acid side chains.

## Results

### Discovery of Aeruginosins

Two abundant peptides with a mass of *m/z* 587 and *m/z* 589 were identified from cell extracts of *Nodularia spumigena* AV1 by LC-MS analysis. They were initially suspected to be new variants of spumigin based on their mass and chromatographic behavior (Figure S1 in File S1). However, fragmentation of the protonated ions *m/z* 587 and *m/z* 589 did not produce sufficient information for overall substructure elucidation and only the presence of argininal and argininol could be postulated (Figures S1–S2 in File S1). Surprisingly, MDA and DNPH derivatization of the compounds and subsequent MS^2^ data suggested that the two peptides contained but differed by the presence of alcohol and aldehyde versions of arginine (Figure S3 in File S1). The moiety is a unique component of the aeruginosin family of linear peptides and we hypothesized that *N. spumigena* make members of the aeruginosin family of peptides.

### Aeruginosin Gene Cluster

**Figure 2:**
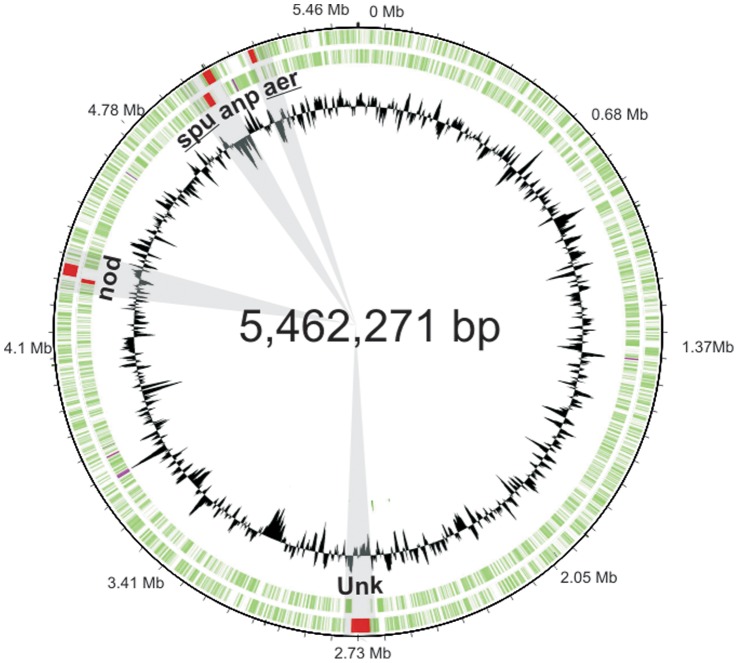
Location of the spumigin and aeruginosin gene clusters on the circular genome of *N. spumigena* CCY9414. The *N. spumigena* CCY9414 genome encodes 5 non-ribosomal peptide gene clusters. The spumigin (*spu*) and aeruginosin (*aer*) gene clusters are encoded 133 kb apart. The spumigin and nodulapeptin (*anp*) gene clusters are encoded 11 kb apart. The nodularin (*nda*) gene cluster and a cryptic NRPS gene cluster (unk) are encoded at separate locations on the chromosome.

A 17.6 kb aeruginosin (*aer*) gene cluster was subsequently identified on a single contig in the genome of *N. spumigena* CCY9414 (GenBank accession number CM001793) through tBLASTn searches using AerD, AerE and AerF protein sequences involved in the synthesis of the Choi moiety (Figure 2). The *aer* gene cluster was located 133 kb apart from the spumigin gene cluster (Figure 2), which was located just 11 kb from the nodulapeptin gene cluster (Figure 2).

**Figure 3:**
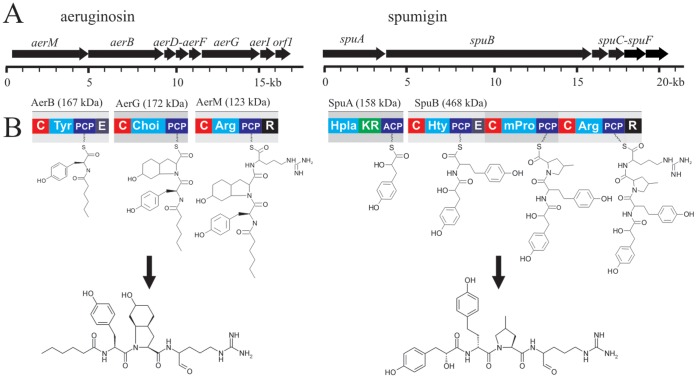
The biosynthesis of aeruginosins and spumigins (A) The organization of the genes in the aeruginosin (18 kb) and spumigin (21 kb) gene clusters in *Nodularia spumigena* CCY9414. (B) The proposed biosynthetic routes and main end-product of each biosynthetic pathway. Adenylation domains and their predicted substrates are given in blue. C, condensation domain; PCP, peptidyl carrier protein; E, epimerase domain; R, reductase domain; KR, ketoreductase domain; ACP, acyl carrier protein.

**Figure 4:**
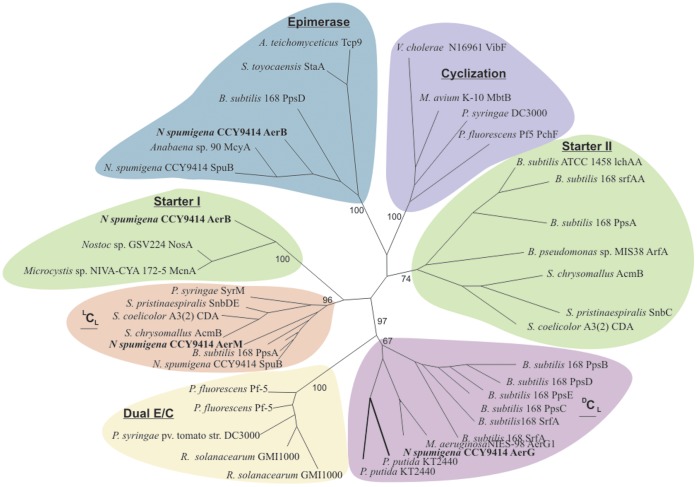
Phylogenetic analyses of the condensation and epimerase domains from the *Nodularia spumigena* CCY9414 aeruginosin gene cluster. The condensation domain of AerB clusters with the starter condensation domains of nostopeptolide (NosA) and cyanopeptolin (McnA) which load short chain fatty acids and forms a well-supported group. The phylogenetic tree includes all condensation subtypes, including condensation (^L^C_L_, ^D^C_L_), condensation and heterocyclization catalyzed by heterocyclization domains, epimerization followed by condensation catalyzed by a Dual E/C domain, and loading condesation domains which are found on initiation modules [29]. The phylogeny was reconstructed using phyml, employing the JTT model of amino acid substitution and a gamma-distributed rate variation with four categories. The support values are based on 100-fold bootstrapping.

The *aer* gene cluster encodes 8 proteins organized in a single operon (Figure 3; Table 1). The predicted substrate specificities of the AerM, AerB and AerG peptide synthetases were L-Arg, L-Tyr and Choi through comparison with other aeruginosin biosynthetic pathways (Table 2). Aeruginosin biosynthesis was predicted to start by loading short-chain fatty acids using the AerB condensation domain, as in a number of other non-ribosomal biosynthetic pathways (Figure 3). In order to test this we performed phylogenetic analyses to assign the condensation domains of AerB, AerM and AerG to different condensation domain subtypes (Figure 4). Phylogenetic analysis of the AerB condensation domain showed a close relationship between the loading condensation domains of the nostopeptolide and cyanopeptolin biosynthetic pathways (Figure 4). Genes encoding the AerD, AerE and AerF enzymes were also located in the *aer* gene cluster (Figure 3) as was a gene encoding a putative glycosyltransferase, AerI (Table 1).

**Table 1:** Proposed functions of genes in the suspected aeruginosin gene cluster.

Protein	Amino acids	Proposed function	Sequence similarity	Organism	Identity (%)	Accession number
ORF	60		MFS-1 protein	*Cyanothece* sp. PCC 8801	43	YP_002373798
ORF	78		MFS-1 protein	*Anabaena* sp. 90	51	YP_006996107
AerM	1469	NRPS	Peptide synthetase	*M. aeruginosa* NIES-843	70	BAG05472
AerB	1451	NRPS	Peptide synthetase	*M. aeruginosa* NIES-843	66	BAG05480
AerD	196	Decarboxylase	AerD protein	*P. rubescens* NIVA-CYA 98	73	CAQ48269
AerE	211	Unknown	AerE protein	*P. agardhii* NIVA-CYA 126	64	CAM59604
AerF	266	Reductase	AerF protein	*M. aeruginosa* NIES-98	83	ACM68688
AerG	1093	NRPS	AerG protein	*M. aeruginosa* NIES-843	75	BAG05474
AerI	133	glycosyltransferase	Glycosyl-transferase	*P. agardhii* NIVA-CYA 126	82	CAM59604
ORF1	259		oxidoreductase	*M. aeruginosa* NIES-98	80	ACM68692
ORF2	394		Signal transduction	*N. punctiforme* PCC 73102	87	ACC84291
ORF3	497		Two-component hybridsensor and regulator	*Nostoc* sp. PCC 7120	87	BAB73236

The assignment of proposed functions to gene products is based upon BLASTp searches.

**Table 2:** The substrate specificity of aeruginosin NRPS adenylation domains.

Protein	Strain	Residue										Proposed substrate
		235	236	239	278	299	301	322	330	331	517	
AerB	CCY 9414	D	A	S	T	I	A	A	V	C	K	Tyr
	NIES-843	–	–	–	–	–	–	–	–	–	–	Tyr
	PCC 7806	–	–	–	–	–	–	–	–	–	–	Tyr
	NIES-98	–	–	F	F	L	G	V	T	F	–	Ile
	CYA126-8	–	–	W	F	L	G	N	–	V	–	Leu
AerG	CCY 9414	D	V	H	I	C	A	F	L	V	K	Choi
	NIES-843	–	–	–	–	–	–	–	–	–	–	Choi
	PCC 7806	–	–	–	–	–	–	Y	–	–	–	Choi
	NIES-98	–	–	–	–	–	–	Y	–	–	–	Choi
	CYA 126-8	–	–	–	–	–	–	–	L	–	–	Choi
AerM	CCY 9414	D	V	E	N	V	G	A	I	T	K	Arg
	NIES-843	–	–	–	–	I	–	–	–	–	–	Arg
	PCC 7806	–	–	–	–	G	A	V	V	–	–	Arg

The aeruginosin producers include *Nodularia spumigena* CCY9414, *Microcystis aeruginosa* strains NIES-843, PCC 7806, NIES-98, and *Planktothrix agardhii* NIVA-CYA 126/8.

**Figure 5:**
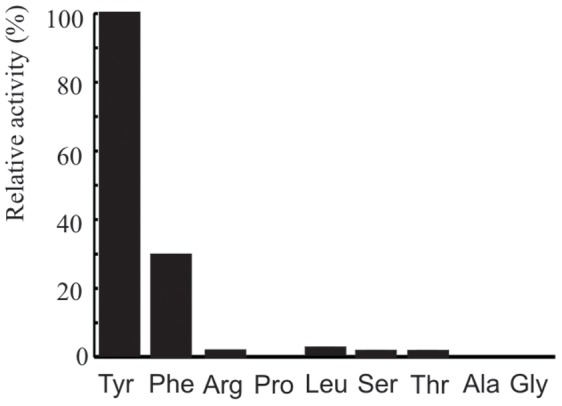
The substrate specificity of the AerB adenylation domain. ATP-PPi exchange assay results for the AerB adenylation domain, showing preferential activation of L-Tyr, a predicted substrate of AerB.

Preliminary MS^2^ fragmentation was not sufficient to resolve the first two sub-structural elements and suggested that the second amino acid could be Leu or Tyr while bioinformatic analyses suggested that the substrate could be L-Tyr (Table 2). In order to gain more information on the substructure of the aeruginosins an ATP-PPi exchange assay was performed which demonstrated that L-Tyr was activated by the AerB adenylation domain *in vitro* (Figure 5). The presence of an epimerase domain suggested that the substrate of AerB was L-Tyr, which would be epimerized to D-Tyr as found in other aeuginosins.

### Aeruginosin Chemical Structure

**Figure 6:**
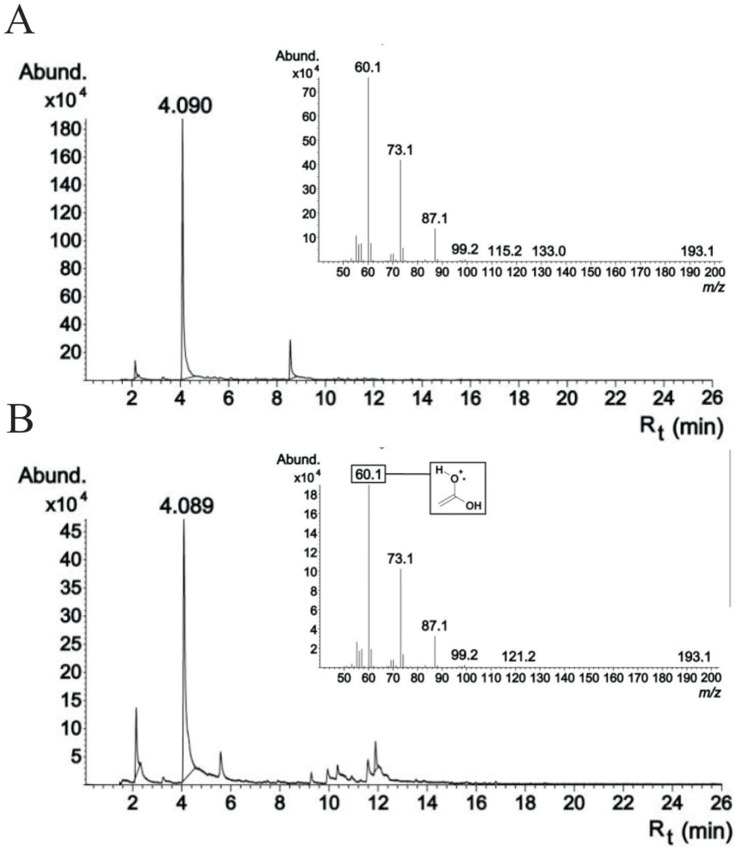
The identity of the C-terminal fatty acid determined using GC-MS. Chromatographic behavior of the hexanoic acid standard and the hydrolysed aeruginosin NOL3. Commercial hexanoic acid and hydrolysis product from NOL3 had identical retention times, 4.089 and 4.090 min, respectively, and identical mass spectra which unambiguously prove the first moiety of the NOL3 aeruginosin. (A) hydrolysed aeruginosin NOL3 (B) hexanoic acid standard.

The main aeruginosin variant (*m/z* 587) was hydrolyzed in order to confirm these biochemical and bioinformatic predictions and to obtain further information on the structure of the new peptides. However, despite the presence of a full-length and apparently functional epimerase domain in AerB, the second amino acid was unambiguously determined to be L-Tyr according to chromatographic amino acid analysis of aeruginosins subjected to acid hydrolysis. The amino acids found at this position in other aeruginosins are almost exclusively D-amino acids. Reanalysis of the main aeruginosin (*m/z* 589) product ion spectrum also showed the presence of Tyr in position 2 (Figure S4 in File S1). The third amino acid, Choi, was in L configuration based to the configuration of Choi in aeruginosin 298-A. The N-terminal moiety could not be detected by amino acid analysis. However, GC-MS analysis of the hydrolyzed aeruginosin allowed unequivocal identification of the N-terminal moiety as hexanoic acid based on retention time and spectra (Figure 6).

The complete structure of the main aeruginosin (*m/z* 587) was confirmed by NMR analysis. Separation and purification of this peptide from the other aeruginosins and spumigins produced by the *N. spumigena* AV1 strain was hindered by the reactive aldehydic nature of the compound (Figure S1 in File S1). NaBH_4_ reduction of the peptides in the methanol extract converted the aldehyde to an alcohol which made it possible to purify reduced aeruginosin by HPLC. ^1^H and ^13^C NMR signals yielded four partial structures confirming the aeruginosin structure (Figure 1A). The NMR spectral data are presented in the supplementary material (Table S1 in File S1; Figures S5–S7 in File S1). Accurate mass measurement of the protonated molecules together with the ^15^N-labeling of the aeruginosins NAL2 and NOL3 were in full agreement with the other results.

### Chemical Variation

**Figure 7:**
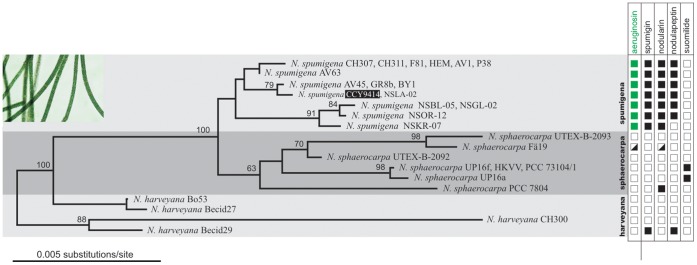
The production of aeruginosins, spumigins, nodularin, nodulapeptin and suomilide by *N. spumigena, N. sphaerocarpa, and N. harveyana*. A maximum-likelihood tree based on the 16S rRNA gene with bootstrap values based on 1000 bootstrap replicates. The taxonomy of the strains follows Lyra et al. 2005 [30] and Lehtimäki et al. 2000 [31]. See also Table S3 in File S1.

Detailed inspection of *N. spumigena* AV1 and CH307 strains allowed the identification of 11 structural variants of aeruginosins (Table 3; Table S2 in File S1). These could be divided into aeruginosins containing either an aldehyde (NAL1-NAL4) or alcohol (NOL1-NOL7) functionality. A range of short straight-chained carboxylic acids were found at the N-terminus (Table 1). Approximately 83% of the variants contained hexanoic acid in AV1 while 93% of the variants contained octanoic acid in CH307 but both strains produced small amounts of aeruginosins with shorter and longer chained carboxylic acids at this position. An *O*-linked pentose was identified in 4 of the 11 aeruginosins and located on the Choi moiety (Figure 1B, Figure S8 in File S1). Inspection of 16 strains of *N. spumigena* revealed just a single strain which lacked detectable levels of aeruginosins (Figure 7 and Table S3 in File S1). Glycosylated aeruginosins could be detected in just 3 of the 16 strains (Table S3 in File S1). Three strains, CH307, CH311 and P38, produced glycosylated aeruginosins, of which CH307 produced variants containing octanoic acid as the main fatty acid.

**Table 3:** Chemical variation of aeruginosins detected from *Nodularia spumigena*.

Aeruginosinvariant	Structural subunits				R_t_	[M+H]^+^ (*m/z*)		Relative amount (%)		
	1	2	3	4	(min)	Experimental^a^	Calculated	AV1	CCY9414	CH307
NAL1	Bu	Tyr	Choi	Argininal	15.0	559.3236	559.3239	1	<1	
NAL2	Hex	Tyr	Choi	Argininal	21.9	587.3553	587.3552	64	66	
NAL3	Oct	Tyr	Choi	Argininal	32.0	615.3877	615.3865	13	1	
NAL4	Oct	Tyr	Choi-P	Argininal	27.8	747				15
NOL1	Ac	Tyr	Choi	Argininol	12.4	533		<1	<1	
NOL2	Bu	Tyr	Choi	Argininol	15.1	561.3381	561.3395	1	1	
NOL3	Hex	Tyr	Choi	Argininol	23.3	589.3708	589.3708	19	29	
NOL4	Oct	Tyr	Choi	Argininol	32.4	617.4050	617.4021	1	<1	
NOL5	Hex	Tyr	Choi-P	Argininol	18.2	721				4
NOL6	Oct	Tyr	Choi-P	Argininol	27.9	749				81
NOL7	Dec	Tyr	Choi-P	Argininol	38.2	777				<1

Structure, ion mass, retention time and abundance of aeruginosin identified from *N. spumigena* AV1, CCY9414 and CH307.

a Unit mass from ion trap, accurate mass from Q-TOF. Ac = acetic acid, Bu = butyric acid,

Hex = hexanoic acid, Oct = octanoic acid, Dec = decanoic acid, P = pentose.

The presence of aeruginosins as well as the distribution of genes encoding AerM, AerB, AerG, and AerI was determined in 16 strains of *N. spumigena*, 4 strains of *N. harveyana*, and 8 strains of *N. sphaerocarpa* (Table S3 in File S1). All 16 strains of *N. spumigena* contained aeruginosin biosynthetic genes and 15 of these strains produced aeruginosins (Figure 7). Aeruginosins were not found from *N. spumigena* AV45, which seems to encode only a partial *aer* gene cluster. All strains of *N. spumigena* from the Baltic Sea contained *aer* clusters encoding the AerI putative glycosyltransferase. The gene encoding this enzyme was not detected in any of the strains isolated from Australia. Neither aeruginosins nor aeruginosin biosynthetic genes could be found in any strains of the benthic *N. harveyana* or *N. sphaerocarpa* tested (Table S3 in File S1). The majority of the 16 strains of *N. spumigena* produced both spumigin and aeruginosin (Figure 7). Some strains produced more aeruginosins and spumigins than others (Figure 7). About half of the 16 strains produced mPro containing spumigins while the remainder produced spumigins containing Pro. Using UV (280 nm) to quantify the amount of aeruginosins in the AV1 strain demonstrated that spumigins comprise 7 ‰ and aeruginosins 3–4 ‰ of the dry weight. All 16 strains of *Nodularia spumigena* produced spumigins and the nodularin toxin. Almost all of the 16 strains of *Nodularia spumigena* produced nodulapeptins. Suomilide was identified only in *N. sphaerocarpa* strains (Table S3 in File S1).

## Discussion

Aeruginosins are a chemically diverse family of peptides known to date from just the bloom-forming cyanobacterial genera *Microcystis* and *Planktothrix* [11], [12], [13], [14]. The relationship between aeruginosins and spumigins has been subject to speculation for some time [12], [13], [15], [16]. Phylogenetic analyses suggest that the spumigin gene cluster of *N. spumigena* and the aeruginosin gene clusters of *Microcystis* and *Planktothrix* are unrelated [8]. Recent studies demonstrated that *N. spumigena* encodes a number of cryptic NRPS clusters for which the products were unknown [3], [17]. It was suggested based on the presence of Choi biosynthetic genes that it may produce an aeruginosin [3]. Interestingly a recent study has shown that *N. spumigena* isolated from variety of geographic locations produce a diversity of peptides including a partial peptide which contains Choi and may be assigned to the aeruginosin family [18]. This study suggests that *N. spumigena* produces *bona fide* aeruginosins in addition to spumigin [18]. However, the aeruginosin structures presented by Mazur-Marzec and coworkers were based on MS^2^ data and are incomplete [18]. The identities of the N-terminal moiety and amino acid at position 2 were not resolved in their analyses [18]. Here we show that the complete structure of the main aeruginosin variant is comprised of an N-terminal short fatty acid chain, L-Tyr, L-Choi and L-argininal.

We found 11 structural variants of the aeruginosins in 16 strains from the Baltic Sea and Australia. The aeruginosins produced by *N. spumigena* contain fatty acids at the N-terminus including acetic acid, butyric acid, hexanoic acid, octanoic acid and decanoic acid. The N-termini of previously reported aeruginosins consists of either hydroxyphenyl lactic acid in *Microcystis* or phenyl lactic acid in *Planktothrix* [11]. Aeruginosins may also be modified to contain chlorine, sulfate or sugars [12] [13]. Glycosylation of aeruginosides, members of the aeruginosin family reported from *P. agardhii,* is catalyzed by the AerI glycosyltransferase [12]. However, the majority of aeruginosins detected here lacked the *O*-linked pentose despite the presence of the AerI glycosyltransferase in the genome of the producing strain.

It has been anticipated that aeruginosins and spumigins might be related compounds based on their structural similarities [12], [13], [15], [16]. Our results show that these two peptides are assembled on separate peptide synthetases (Figure 2). The organization of catalytic domains in AerB, AerG and AerM suggests an orthodox model for aeruginosin assembly (Figure 2). *N. spumigena* CCY9414 lacks the reductive loading mechanism of the *Microcystis* and *Planktothix* aeruginosin biosynthetic pathways entirely [12], [13]. The organization of catalytic domains in the *aer* gene cluster (Figure 3) and phylogenetic analyses (Figure 4) suggests that the condensation domain of AerB is responsible for lipidation of the aeruginosins. N-terminal condensation domains have been proposed to prime the synthetase with short-chain carboxylic acids in lichenysin [19], daptomycin [20], nostopeptolide [21] and cyanopeptolin [22] [23] biosynthesis. Our results suggest substrate specificities ranging from C_2_ to C_10_ fatty acid moieties. However, the exact lipidation mechanism remains unknown. The reductase domain of AerM releases the C-terminal arginine as a reactive aldehyde.

Members of the aeruginosin family of natural products commonly have strong inhibitory activity against serine proteases [11], [15]. Serine proteases are involved in a number of important physiological processes, and their importance in the complex blood coagulation cascade is well established [11]. Planktonic bloom-forming cyanobacteria produce a range of protease inhibitors [15]. The function of these peptides is unclear but they are widely believed to be part of a chemical defense system, acting as a grazing deterrent [24], [25]. Our results show that *N. spumigena* strains produce a complex cocktail of protease inhibitors comprising up to 1% of the dry weight of the organism, which may explain in part its ecological success.

## Materials and Methods

### Strain Growth

Sixteen strains of *N. spumigena*, 4 strains of *N. harveyana* and 8 strains of *N. sphaerocarpa* (Table S3 in File S1) were grown at a photon irradiance of 15 µmol m^−2^ s^−1^ in saline Z8 medium lacking a source of combined nitrogen for 21 days [8]. ^15^N-labeling of *N. spumigena* AV1 was performed in similar way, except that medium was buffered with 10 mM HEPES (pH 8.0). ^15^N-urea (98+ % ^15^N, ISOTEC, USA) was used as nitrogen source and nitrogen-free argon (with 20.9% O_2_ and 0.45% CO_2_; quality 5.7; AGA Gas Ab, Sweden) was bubbled into the medium to prevent nitrogen fixation from air.

### Gene Cluster Annotation

The *aer* gene cluster was identified on a single 5,462,271 bp scaffold in the genome of *N. spumigena* CCY9414 (GenBank accession number CM001793) through BLASTp searches using AerD, AerE and AerF proteins. The genes in the *aer* gene cluster were predicted with Artemis using Glimmer. The starting sites were refined manually. The amino acid sequences of the genes were used to query the non-redundant database at NCBI in order to predict a function for the genes (Table 2). The substrate specificity of the activated adenylation domains in the NRPS modules was predicted by using the 10 amino acid binding pocket signature [26].

### Frequency of *aer* gene Clusters in *N. spumigena* Strains

Genomic DNA was extracted from the cultivated strains as previously described [8]. We amplified four genes from the *aer* gene cluster, *aerM*, *aerB*, *aerG* and *aerI*, by PCR using oligonucleotide primers designed from the *N. spumigena* CCY9414 genome sequence (Table S4 in File S1). The PCR reactions were performed in a 20 µl final volume containing 1 µl of DNA, 1× DyNAzyme II PCR buffer, 100 mM of each deoxynucleotide, 0.4 mM of each oligonucleotide primer, and 0.4 units of DyNAzyme II DNA polymerase (Finnzymes, Espoo, Finland). The following protocol was used: 94°C, 3 min; 25 cycles of 94°C, 30 s; 63°C, 30 s; 72°C, 1 min; and 72°C, 10 min. PCR to confirm the deletion of the *aerI* gene was performed as before but with an annealing temperature of 58°C. PCR products were visualized on 1.5% agarose gels containing 0.5× TAE run at 120 V for 20–25 min and scored for the presence or absence of PCR products of the expected length. The 16S rRNA gene was amplified and sequenced from *N. spumigena* CH307, P38 and AV45 as previously described [27] and the sequence data was deposited in GenBank (KF360086-KF360088). An alignment of 16 strains of *N. spumigena*, 8 strains of *N. sphaerocarpa* and 4 strains of *N. harveyana* was made using Bioedit. Gaps and ambiguous regions were excluded and a total of 1340 bp of sequence was considered for phylogenetic analysis. A neighbor-joining tree was constructed using DNADIST and NEIGHBOR as implemented in the PHYLIP package. The tree was midpoint rooted using RETREE. 1000 bootstrap replicates were constructed using SEQBOOT, DNADIST, NEIGHBOR and CONSENSE in the PHYLIP package. The production of aeruginosin, spumigin, nodularin, nodulaopeptin and suomilide was mapped to this tree.

### LC-MS

LC-MS analyses were performed with an Agilent 1100 Series LC/MSD Ion Trap XCT Plus System (Agilent Technologies, Palo Alto, CA, USA) using a Phenomenex Luna C8 (150×2.0 mm, 5 µm, Phenomenex, Torrance, CA, USA) LC-column. Between 7 and 31 mg of freeze-dried cells of *Nodularia* strains were extracted for 20 s with 1 ml of methanol in 2 ml plastic tubes containing approximately 200 µl of 0.5 mm glass beads (Scientific Industries, New York) using Fast Prep homogenizer (FP120, Bio 101, Savant) at speed value of 6 m s^−1^. Extracts were centrifuged for 5 min at 10 000 *g* prior to LC-MS analysis. High accuracy mass of the aeruginosins of *N. spumigena* AV1 was measured by UPLC-ESI-QTOF mass spectrometry performed on Synapt G2 HDMS (Waters, MA, USA) in high resolution mode and *m/z* 500–850 mass range. In MS/MS analysis the mass range was *m/z* 50–531.

### Derivatization

Aeruginosins were derivatized with malondialdehyde (MDA) using 100 µl of methanol extract from *N. spumigena* AV1 evaporated to dryness in vacuum centrifuge. The resultant residue was dissolved in 100 µl of 12 M H_3_PO_4_ and 2.4 µl of 1,1,3,3-tetraethoxypropane (Sigma) was added. The sample was evaporated in a vacuum centrifuge after 1 h at room temperature and dissolved in 100 µl of methanol. DNPH derivatives were prepared as previously described [8].

### Amino Acid Hydrolysis

Isolated aeruginosin NOL3 (100 µg) were dried in a 300-µl glass vial which was then transferred to a 4-ml glass vial containing 1 ml of 6 M HCl. The vial was flushed with argon prior to being closed. Acid hydrolysis was performed by incubating overnight at 110°C. After hydrolysis, the inner vial was dried for 30 min with a vacuum centrifuge. Hydrolyzed and reference amino acids were derivatized by the Marfey method using L-FDAA (1-fluoro-2,4-dinitrophenyl-5)-L-alaninamide) reagent. Reaction mixtures were analyzed with a Luna C18(2) column (150×2, 5 µm; Phenomenex) eluted with an acetonitrile (solvent B) and 0.1% aqueous formic acid (solvent A) gradient (20% B to 75% B in 45 min and then 5 min at the reached level) at a flow rate of 0.2 ml min^−1^ at a temperature of 40°C. The detection wavelength for the Marfey derivatives was 340 nm. Acid hydrolysate of aeruginosin 298-A from *Microcystis aeruginosa* NIES-298 was used as a reference for L-Choi [28].

### GC-MS

100 µg of aeruginosin NOL3 was incubated with 100 µl of 5 M NaOH in a closed vial for 24 h at 110°C. The entire 100 µl solution was transferred to a 20 ml brown vial with 18 mm magnetic caps with silicon/Teflon disks (VWR International, USA) containing 4 ml of MilliQ water, 1.5 g of NaCl and 50 µl of 17.5 M H_3_PO_4,_ and the vial was immediately sealed. The vial was agitated for 5 min at 70°C and 500 rpm in a GC-MS autosampler (combiPAL, CTC Analytics). A SPME (Solid Phase Micro Extraction) needle penetrated 12 mm through the vial cap, exposing 10 mm of the fiber (DVB/CAR/PDMS, Supelco, Sigma-Aldrich Co., USA) inside the vial. After a 30 min extraction the fiber was retracted and sample was injected in the GC column with an injection needle penetration of 32 mm and the fiber exposure of 10 mm. After 10 min of desorption time, the fiber was removed from the injection port and the sample was injected with a split ratio of 5∶1. HP 6890 gas chromatograph with an Agilent 5973 Network mass selective detector (Agilent Technologies, Wilmington, DE, USA) with a split/splitless injector and a SPB™-624 capillary column (30 m×0.25 mm, 1.4 um; Supelco, Sigma-Aldrich Co., USA) were used as follows: Injection and detector at 250°C, oven started at 150°C for 2 min from which temperature increased 5°C min^−1^ during 10 min. The final temperature was 220°C after a total run time of 26 min. Helium was used as carrier gas with a flow rate of 1 ml min^−1^. A standard 0.14 mM hexanoic acid (Sigma-Aldrich Co., USA) solution was prepared. The full scan electron impact mass spectra were obtained at a range of 50–200 m/z.

### NMR Analysis

Aldehydes were converted to alcohols in the methanol extract using NaBH_4_ reduction which made it possible to purify aeruginosin NOL1 by HPLC. One gram of freeze dried AV1 cells was extracted with 70 ml of methanol using a tip homogenizer (SilentCrusher M, Heidolph, Germany) in three 30 sec cycles at ambient temperature with a speed of 16000 rpm 3×30 sec. The suspension was centrifuged (10000 g, 5 min) and dichloromethane and water was added to the supernatant in volume ratio of 1∶1:1. Phases were separated by centrifugation (5000 g, 5 min). The upper water/methanol phase was collected and vacuum evaporated to dryness. The residue was dissolved in 4 ml of methanol, 50 mg of NaBH_4_ was added and after 5 min reaction time the solution was vacuum evaporated to dryness. The residue was dissolved in 1 ml of 15% acetonitrile. 100 µl portions of the solution were injected 10 times into a Luna C8 (2) (10×150 mm, 5 µm, 100 Å, Phenomenex) column which was eluted isocratically with 0.1% TFA in 15% acetonitrile. Pooled fractions containing aeruginosin NOL1 were evaporated in a vacuum and dissolved in CD_3_OD for NMR. ^1^H and ^13^C NMR spectra were obtained with a Varian Unity Inova 600 MHz NMR spectrometer equipped with cryogenically cooled triple-resonance ^1^H, ^13^C, ^15^N probe head and actively shielded z-gradient system. DQF-COSY, TOCSY (120 ms mixing time) experiments were collected using 2048 and 512 data points in F_1_ and F_2_ dimensions, corresponding to acquisition times of 0.34 and 0.085 s, respectively. The corresponding acquisition times in ^13^C HSQC and ^13^C HMBC experiments were 0.02 (^13^C dimension) and 0.17 (^1^H dimension), and 0.014 (^13^C dimension) and 0.34 (^1^H dimension), respectively. The average one- and three-bond ^1^H-^13^C couplings were estimated to be 140 Hz and 8 Hz, and ^1^H-^13^C transfer delays for HSQC and HMBC were set to 3.57 and 62.5 ms, respectively. All spectra were collected at 25°C. Spectra were processed and analyzed using VNMRJ 2.1 version B and ACD/SpecManager version 11.03 software packages.

### ATP-pyrophosphate Exchange Assay

The region of the *aerB* gene encoding the adenylation domain was amplified by PCR from *N. spumigena* CCY9414 using oligonucleotide primers designed to anneal to the substrate-conferring portion of each adenylation domain. Primer design and PCR reactions were performed as described previously [8]. PCR products were digested with *Nco*I and *Pme*I restriction enzymes, gel excised and ligated to pFN18A (HaloTag® 7) T7 Flexi® vector (Promega, WI, USA) opened with the same enzymes. Ligation mix was transformed into *Escherichia coli* (KRX) competent cells following the manufacturer’s instructions. Colonies were grown in shaker (160 rpm) at 37°C overnight in 3 ml of LB medium supplemented with 100 mg ml^−1^ ampicillin. In the following day, 400 µl was used to inoculate 20 ml of TB medium containing 50 mg ml^−1^ carbenicillin and incubated with shaking at 37°C (160 rpm) for 1.5 h and then induced by the addition of 0.1% of rhamnose and the culture was grown overnight (16–18 h) in shaker (100 rpm) at 24°C. *E*. *coli* cells were collected and sonicated as described previously [8]. The expression of soluble protein was observed in 10% SDS PAGE gel. The soluble adenylation domains were purified using HaloTag® Protein Purification System (Promega). Protein concentration of the preparations was measured with the BCA protein assay kit (Pierce). ATP-pyrophosphate exchange assay was performed as described previously [9].

## Supporting Information

File S1 The Combined Supporting Information File S1 contains detailed data on the discovery and identification of aeruginosins by LC-MS (Figures S1–S4) and NMR (Figures S5–S7, Table S1), chemical variation of aeruginosins (Figure S8 and Table S2) and the results of the screening of individual *Nodularia* strains for various peptides and their biosynthetic genes (Table S3) and the PCR primers used (Table S4).
(DOCX)
