# Supplementary material for: New Structural Variants of Aeruginosin Produced by the Toxic Bloom Forming Cyanobacterium Nodularia spumigena
Source: PLoS One. 2013 Sep 6;8(9):e73618. doi: 10.1371/journal.pone.0073618 (PMC3765200; doi:10.1371/journal.pone.0073618)
Supplement: File S1 — The Combined Supporting Information File S1 contains detailed data on the discovery and identification of aeruginosins by LC-MS (Figures S1–S4) and NMR (Figures S5–S7, Table S1), chemical variation of aeruginosins (Figure S8 and Table S2) and the results of the screening of individual Nodularia strains for various peptides and their biosynthetic genes (Table S3) and the PCR primers used (Table S4). (DOCX) [file pone.0073618.s001.docx]

Supporting Information

**The Combined Supporting Information File S1**

**Discovery of aeruginosins**

During LC-MS analysis of *Nodularia* peptides an abundant compound pair of m/z 587 and m/z 589 (MH+) was found the chromatographic behaviors of which were similar to that of spumigins (Fig. S1). However, the structure of these new peptides could not be solved because fragmentation of the protonated ions m/z 587 and m/z 589 did not produce a sufficient number of ions for substructure elucidation. Only the presence of either argininal or argininol could be postulated from the neutral loss of 42 Da (HN=C=NH) characteristic for guanidine group of the arginine (Fig. S2). Reaction of the m/z 587 compound with dinitrophenylhydrazine and the fragments from the DNPH-derivative ion m/z 767 proved the presence of argininal but not the other substructures (Fig. S3). In order to obtain new structurally informative ions the fragmentation mechanism of the unknown compounds was changed by malondialdehyde (MDA) derivatization [1]. MDA reacts with high proton affinity to the guanidine group of the argininal/argininol residue and changes it to a less basic pyrimidine ring. This modification increases proton mobility and in principle increases the number of structurally relevant product ions. LC-MS analysis of the derivatized methanol extract showed that all formed MDA-derivatives were in alcohol form. Comparison of the product ion spectra of MDA-spumigin A and MDA-derivative from the unknown compounds m/z 587 and m/z 589 (MH+) gave clear indication that these compounds contain the rare Choi moiety instead of Mpr or Pro (Figure S3). However, the remaining structural elements could not be identified.

The tentative finding of Choi led to the discovery of a NRPS gene cluster with 3 modules from *N. spumigena* CCY9414 which code the synthesis of a peptide with a constituent amino acid Choi. Based to these findings the unknown compounds were not the typical *Nodularia* tetrapeptide spumigins but aeruginosins and were accordingly named aeruginosin NAL1 (m/z 587) and NOL1 (m/z 589). Tyrosine was the most preferred substrate in substrate specificity analysis of the AerM adenylation domain. Reanalysis of the product ion spectrum of aeruginosin NOL1 also showed the possible presence of tyrosine probably in position 2 based on the similar fragmentation behavior of protonated ions of aeruginosin NOL1 and spumigin A (Figure S4). This interpretation of aeruginosin NOL1 product ion spectra gave a mass of 98 Da for the fourth substructure in position 1 but the structure itself remained unsolved.

According to chromatographic amino acid analysis of the acid hydrolyzed aeruginosin NOL1 the variable position 2 amino acid was unambiguously L-Tyr. Most of the position 2 amino acids have been reported to be D-amino acids. In case of aeruginosin 298-A and B position 2 amino acids was originally interpreted to be L-Leu but was later shown to be D-Leu according to chemical synthesis [2].





**Figure S1.** Extracted ion chromatograms of spumigins E (MH^+^ = *m/z* 611) and A (MH^+^ = *m/z* 613) and the unknown compounds with MH^+^ of *m/z* 587 and *m/z* 589.





**Figure S2**. Product ion spectra of the protonated spumigins E and A, and the unknown compounds with MH^+^ of *m/z* 587 and *m/z* 589. Neutral loss of 42 Da (HN=C=NH) indicates the presence of a guanidino group, a structure in Arg side chain.





**Figure S3**. Product ion spectra of the DNPH- and MDA-derivatives of the protonated molecules of spumigin E, A, and the unknown compounds with MH^+^ of *m/z* 587 and *m/z* 589. The structure of DNPH-spumigin E is described in the uppermost panel and the MDA derivatization reaction in the third panel. DNPH, 2,4-dinitrophenylhydrazine; mPro, 4-methylproline; HPLA, (4-hydroxy)phenyllactic acid; Hty, homotyrosine; APAP, 2-amino-5-(pyrimidin-2-ylamino)pentan-1-ol; Choi, 2-carboxy-6-hydroxyoctahydroindole.





**Figure S4.** Product ion spectra of aeruginosin NOL3 (MH^+^ = *m/z* 589) and protonated spumigin A.

**NMR**

The complete structure of novel NOL3 aeruginosin was solved with NMR analysis. Separation and purification of the native NAL2 aeruginosin from the chemically related compounds produced by the *Nodularia spumigena* AV1 was hindered by the reactive aldehydic nature of the compounds. NaBH_4_ reduction of the methanol extract converted aldehydes to alcohols which made possible HPLC purification of the reduced aeruginosin NAL2. ^1^H NMR (Fig. S5), ^1^H-^1^H DQF-COSY, ^1^H-^1^H TOCSY, ^13^C gHSQC and ^13^C gHMBC spectra (Fig. S6) revealed four partial structures as expected which were Hex (hexanoic acid, X in Fig. S4), Tyr, Choi (2-carboxy-6-hydroxyoctahydroindole) and Arg-OH (argininol) all presented in Table S1. Signal δ 0.87(t) assigned as a terminal methyl in alkyl chain showed TOCSY correlations with two signals δ 1.17 and 1.27 typical for saturated alkyl chain methylene protons, with a signal δ 1.49 typical for acyl C3 methylene protons and with a signal δ 2.13 typical for acyl C2 methylene protons (Fig. S6, A). HSQC connected aforementioned protons to carbons with signals typical for acyl chain carbons (Fig. S6, B). The methyl and methylene group connectivities from COSY (Fig. S6, C) together with HBMC correlations of C2 and C3 methylene protons to a carbonyl (δ 175.8) (Fig. S6, D) finalized this partial structure to be hexanoic acid (Hex). Hex proton and carbon signals except H4/C4 signals were in excellent agreement to signals reported earlier for hexanoic acid connected to a amide nitrogen [3], [4] ,[5] [6]. The mass of hexanoic acid without water is 98 Da and thus same with the mass of the unknown neutral fragment X formed from the protonated aeruginosin NOL3 (Figure S4). As H2^Tyr^ (δ 4.71) had strong correlation with the carbonyl carbon of Hex (Fig. S6, D), hexanoic acid is attached to the amino group of tyrosine and form the position 1 substructure.

Diagnostic 1D proton signal patterns for a *p*-oxygensubstituted phenyl ring was observed at δ 6.73 and 7.10 (Fig. S6) and corresponding carbons at δ 116.2 and 131.3 (Fig. S6, B). Aromatic oxygen-bearing carbon signal at δ 157.3, carbonyl carbon signal at δ 172.8 and other typical proton and carbon signal correlations in HMBC and COSY spectra (Fig. S6, E, F) proved the partial structure in position 2 to be Tyr.

In addition of H2 and H3,3’ correlations with C1 in Tyr, two other protons δ 4.37 and 4.67 with carbons δ 62.1 and 56.0, respectively, also had correlations to C1^Tyr^ (Fig. S6, D). These protons formed a spin system with several other protons with 7 resolved signals (from δ 1.56 to 2.41) at aliphatic region and one signal δ 4.03 referring to a proton next to a electronegative atom (Fig. S6, G). These results together with corresponding carbon signals from HSQC (Fig. S6, B) and with correlations from COSY (Fig. S6, H) and HMBC (Fig. S6, E, I) showed that this partial structure in position 3 was Choi, amino acid unique for aeruginosins. Chemical shifts measured in CD_3_OD (Table S1) were somewhat different than those reported in literature and measured in DMSO-d_6_ [7], [8] or in CD_3_OD from acetylated aeruginosin 298-A (9). Chemical shifts δ 4.4 and 34.7 of H7a and C7 were more analogous with corresponding signals from microcin SF608 and synthetic aeruginosin 298-A (1a) which contain L-amino acid next to Choi (position 2) than the signals from all other aeruginosins with L-amino acid in the position 2 [4], [10]. This result is consistent with the amino acid analysis which revealed exclusively Tyr amino acid in L configuration from the aeruginosin NOL3 hydrolysate.

The last partial structure (position 4) could be recognized from the correlation of proton δ 3.90 with CO^Choi^ signal of δ 174.2 (Fig. S6, D). Proton δ 3.90 formed a spin system with protons represented by 2 signals at aliphatic region (δ 1.5 - 1.8) and two signals at region (δ 3.2 - 3.5) referring to protons next to a electronegative atom (Fig. S6, J). These proton signals were directly connected to five carbon atoms (Fig. S6, B). As proton δ 3.24 showed correlation to a carbon at δ 158.5 which signal is characteristic for a guanidine quaternary carbon and no other quaternary carbon correlations existed (Fig. S6, D, E) this partial structure was Arg-OH (argininol). DQF-COSY correlations of all subunits are summarized in Fig. S7 together with the HMBC correlations linking the subunits together.

**Table S1.** NMR data of aeruginosin NOL3 in CD_3_OD.

| Unit | C/H No | δC (ppm) | δH (ppm) | HMBC^a^ | |
| --- | --- | --- | --- | --- | --- |
| Hex | 1 | 175.8 |  |  | |
|  | 2 | 36.1 | 2.13 | Hex 1, 3, 4 | |
|  | 3 | 26.3 | 1.49 | Hex 1, 2, 4, 5 | |
|  | 4 | 31.9 | 1.17 | Hex 2, 3, 6 | |
|  | 5 | 23.1 | 1.27 | Hex 3, 4, [5], 6 | |
|  | 6 | 13.8 | 0.87 | Hex 4, 5 , [6] | |
| Tyr | 1 | 172.8 |  |  | |
|  | 2 | 53.7 | 4.71 | Tyr 1, 3, 4; Hex 1 | |
|  | 3 | 37.8 | 2.77 | Tyr 1, 2, 4, 5/9 | |
|  | 3' |  | 3.00 | Tyr 1, 2, 4, 5/9 | |
|  | 4 | 128.6 |  |  | |
|  | 5/9 | 131.3 | 7.10 | Tyr 3, 6/8, 7 | |
|  | 6/8 | 116.2 | 6.73 | Tyr (4), (5/9), [6/8], 7 | |
|  | 7 | 157.3 |  |  | |
| Choi | 1 | 174.2 |  |  | |
|  | 2 | 62.1 | 4.37 | Choi 1 , 3, (7a); Tyr 1 | |
|  | 3 | 31.6 | 2.05 | Choi 1, 2, 3a, 4 | |
|  | 3' |  | 2.09 | Choi 3a, 7a | |
|  | 3a | 37.8 | 2.41 |  | |
|  | 4 | 19.7 | 1.56 |  | |
|  | 4' |  | 2.17 |  | |
|  | 5 | 26.3 | 1.58 |  | |
|  | 6 | 66.4 | 4.03 |  | |
|  | 7 | 34.7 | 1.82 | Choi 3a, 7a, 5, 6, 7a | |
|  | 7a | 56.0 | 4.67 | Choi 2, 3a; Tyr 1 | |
| Arg-OH | 1 | 64.7 | 3.50 | Arg-OH 2, 3 | |
|  | 1' |  | 3.52 |  | |
|  | 2 | 51.5 | 3.90 | Arg-OH (1), 3, 4; Choi 1 | |
|  | 3 | 29.1 | 1.51 | Arg-OH 1, 2, 4, 5 | |
|  | 3' |  | 1.73 | Arg-OH 1, 2, 4, 5 | |
|  | 4 | 25.7 | 1.74 | Arg-OH 1, 2, 3, 5 | |
|  | 5 | 41.8 | 3.24 | Arg-OH 3, 4, 7 | |
|  | 7 | 158.5 |  |  | |
| [H..] = Split signal, (H..) = Weak signal  ^a^HMBC correlations are from the proton to the carbon. | | | | |  |

**

**

**Figure S5.** A) ^1^H NMR spectrum of aeruginosin NOL3; B) low field range of the spectrum.

**Figure S6.** Annotated 2D NMR spectra of aeruginosin NOL3. **A:** ^1^H-^1^H-TOCSY signals of Hex protons.

7.10 7.00 6.90 6.80 6.70

118.0

122.0

126.0

130.0

**Figure S6.** Annotated 2D NMR spectra of aeruginosin NOL3. **B:** ^13^C gHSQC spectrum with aromatic Tyr signals inside the insertion.

**Figure S6.** Annotated 2D NMR spectra of aeruginosin NOL3. **C:** ^1^H-1H DQF-COSY signals of Hex protons.

**Figure S6.** Annotated 2D NMR spectra of aeruginosin NOL3. **D:** ^13^C gHMBC signals of carbonyl carbons.

**Figure S6.** Annotated 2D NMR spectra of aeruginosin NOL3. **E:** ^13^C gHMBC spectrum.

**Figure S6.** Annotated 2D NMR spectra of aeruginosin NOL3. **F:** ^1^H-1H DQF-COSY signals of Tyr protons.

**Figure S6.** Annotated 2D NMR spectra of aeruginosin NOL3. **G:** ^1^H-^1^H-TOCSY signals of Choi protons

**Figure S6.** Annotated 2D NMR spectra of aeruginosin NOL3. **H:** ^1^H-1H DQF-COSY signals of Choi protons.

**Figure S6.** Annotated 2D NMR spectra of aeruginosin NOL3. **I:** ^13^C gHMBC high field spectrum

**Figure S6.** Annotated 2D NMR spectra of aeruginosin NOL3. **J:** ^1^H-^1^H-TOCSY signals of Arg-OH protons

**Figure S6.** Annotated 2D NMR spectra of aeruginosin NOL3. **K:** ^1^H-1H DQF-COSY signals of Arg-OH protons.





**Figure S7.** Structure of aeruginosin NOL3 with ^1^H-^1^H DQF-COSY correlations (bold lines) linking atoms next to each other together and ^13^C HMBC (arrows) correlations linking substructures/spin units to each other.

Chemical variation of aeruginosins

The presence of pentose in aeruginosin NAL4, NOL5, NOL6 and NOL7 variants was based on the loss of neutral fragment 132 Da from protonated molecular ions in mass spectrometric analysis (Fig. S8). O-linked monosaccharides typically detach from the aglycone as a dehydrated residues which mass is 132 Da in case of pentose sugar. The position of the pentose moiety in the aeruginosin variants was deduced from the product ion spectra of the protonated ions of the NOL variants. Spectra of the most abundant NOL variants are presented in Figs. S4 and S8. All Choi containing NOL variants formed stable product ions *m/z* 328 [Choi–Arg-OH+H]^+^ and the base peak *m/z* 311 (328-NH_3_). Additionally NOL variants with pentose moiety produced pentose containing product ions *m/z* 460 [Choi-pentose–Arg-OH+H]^+^ as a base peak and intense *m/z* 443 (460-NH_3_). As pentose was found also from the variant NAL4 where the only hydroxyl in the Choi–argininal part of the molecule is in Choi, the only location where an O-linked pentose could situate is Choi.

**

**

**Figure S8.** Product ion spectrum of glycosylated aeruginosin NOL6 (MH^+^ = *m/z* 749). Oct = Octanoic acid.

**Table S2.** Aeruginosin variants, ion masses of native and ^15^N labeled aeruginosins, calculated number of nitrogen atoms in the variant molecules, exact and calculated masses and error in ppm of aeruginosins identified from *Nodularia spumigena* AV1.

|  |  |  |  |  |  | Ion trap measurement | | |  | Accurate mass measurement | | |
| --- | --- | --- | --- | --- | --- | --- | --- | --- | --- | --- | --- | --- |
| Aeruginosin | Structural subunits | | | |  | [M+H]^+^ (*m/z*) | | Number |  | Experimental | Calculated | Error |
| variant | 1 | 2 | 3 | 4 |  | ^14^N | ^15^N | of N |  | mass | mass | (ppm) |
| NAL1 | Bu | Tyr | Choi | Argininal |  | 559 | 565 | 6 |  | 559.3236 | 559.3239 | -0.40 |
| NAL2 | Hex | Tyr | Choi | Argininal |  | 587 | 593 | 6 |  | 587.3553 | 587.3552 | 0.28 |
| NAL3 | Oct | Tyr | Choi | Argininal |  | 615 | 621 | 6 |  | 615.3877 | 615.3865 | 2.06 |
|  |  |  |  |  |  |  |  |  |  |  |  |  |
| NOL2 | Bu | Tyr | Choi | Argininol |  | 561 | 567 | 6 |  | 561.3381 | 561.3395 | -2.56 |
| NOL3 | Hex | Tyr | Choi | Argininol |  | 589 | 595 | 6 |  | 589.3708 | 589.3708 | -0.07 |
| NOL4 | Oct | Tyr | Choi | Argininol |  | 617 | 623 | 6 |  | 617.4050 | 617.4021 | 4.68 |

**Screening results**

16 strains of *N. spumigena*, 8 strains of *N. sphaerocarpa*, 4 strains *N. harveayana* were screened for the presence of the aeruginosin gene cluster as well as the cyclic and linear peptide profiles. These strains were assigned to these species of *Nodularia* based on their morphology and confirmed by 16S rRNA gene sequence data [12], [13]. The primers were designed to amplify 4 genes involved in the biosynthesis of aeruginosin, *aerM*, *aerB*, *aerG* and *aerI* (Table S6). The strains were examined (Table S5) for the presence or absence of aeruginosin, nodularins, spumigins, nodulapeptins and suomilides as previously described (11). The peptides which were examined are as follows: Nodularin-R, [D-Asp^1^]Nodularin; Spumigins A, D, E, F, G, H Nodulapeptin A, B and variants with unknown structures and [M+H]^+^ 's of 778, 794, 808 and 902; Suomilide and variants with unknown structures and [M+H]^+^ 's of 852, 922, 950, 1020, 1064 and 1076; Aeruginosins according this publication.

**Table S3** 16 strains of *N. spumigena*, 8 strains of *N. sphaerocarpa*, 4 strains *N. harveayana*. The PCR for the studied genes was performed using the primer pairs aep1, aep2, aep6, aep7 and aep68 (Table S6).

| Species ^a^ | | Strain | 16S rRNA | Origin | *aer* gene cluster | | | | | Peptide families | | | | |
| --- | --- | --- | --- | --- | --- | --- | --- | --- | --- | --- | --- | --- | --- | --- |
|  |  |  |  |  |  |  |  |  |  | Nodularins | Spumigins | Aeruginosins | Nodulapeptins | Suomilides |
|  |  |  |  |  | *aerM* | *aerB* | *aerG* | *aerI* | *aerI* |  |  |  |  |  |
| *N. spumigena* | | GR8b | AJ133178 | Baltic | **+** | **+** | **+** | **+** | **+** | ● | ● | ● | ● | **–** |
| *N. spumigena* | | AV1 | AJ781136 | Baltic | + | + | + | + | + | ● | ● | ● | ● | **–** |
| *N. spumigena* | | F81 | AJ781137 | Baltic | **+** | **+** | **+** | **+** | **+** | ● | ● | ● | ● | **–** |
| *N. spumigena* | | BY1 | AF268004 | Baltic | **+** | **+** | **+** | **+** | **+** | ● | ● | ● | ● | **–** |
| *N. spumigena* | | CH311 | AJ781133 | Baltic | **+** | **+** | **+** | **+** | **+** | ● | ● | ● ^b^ | ● | **–** |
| *N. spumigena* | | CCY9414 | CM001793 | Baltic | **+** | **+** | **+** | **+** | **+** | ● | ● | ● | ● | **–** |
| *N. spumigena* | | AV45 | KF360088 | Baltic | **+** | **+** | **-** | **-** | **-** | ● | ● | **–** | ● | **–** |
| *N. spumigena* | | AV63 | AJ781138 | Baltic | **+** | **+** | **+** | **+** | **+** | ● | ● | ● | ● | **–** |
| *N. spumigena* | | CH307 | KF360086 | Baltic | **+** | **+** | **+** | **+** | **+** | ● | ● | ● ^b^ | **–** | **–** |
| *N. spumigena* | | P38 | KF360087 | Baltic | **+** | **+** | **+** | **+** | **+** | ● | ● | ● ^b^ | ● | **–** |
| *N. spumigena* | | HEM | AJ781134 | Baltic | **+** | **+** | **+** | **+** | **+** | ● | ● | ● | ● | **–** |
| *N. spumigena* | | NSLA-02-a | AF268008 | AUS | **+** | **+** | **+** | **-** | **d^c^** | ● | ● | ● | ● | **–** |
| *N. spumigena* | | NSBL-05 | AF268012 | AUS | **-** | **-** | **+** | **-** | **d** | ● | ● | ● | ● | **–** |
| *N. spumigena* | | NSGL-02 | AF268015 | AUS | **+** | **+** | **+** | **-** | **d** | ● | ● | ● | ● | **–** |
| *N. spumigena* | | NSOR-12 | AF268011 | AUS | **+** | **+** | **+** | **-** | **d** | ● | ● | ● | ● | **–** |
| *N. spumigena* | | NSKR-07 | AF268013 | AUS | **+** | **+** | **+** | **-** | **d** | ● | ● | ● | **–** | **–** |
| *N.* *sphaerocarpa* | | Fä19 | AJ781144 | Baltic | **-** | **-** | **-** | **-** | **-** | (●) | **–** | (●) | **–** | **–** |
| *N. sphaerocarpa* | | UP16f | AJ133182 | Baltic | **-** | **-** | **-** | **-** | **-** | **–** | **–** | **–** | **–** | ● |
| *N. sphaerocarpa* | | HKVV | AJ133183 | Baltic | **-** | **-** | **-** | **-** | **-** | **–** | **–** | **–** | **–** | ● |
| *N. sphaerocarpa* | | UP16a | AJ781140 | Baltic | **-** | **-** | **-** | **-** | **-** | **–** | **–** | **–** | **–** | ● |
| *N. sphaerocarpa* | | PCC73104/1 | AJ781139 | CAN | **-** | **-** | **-** | **-** | **-** | **–** | **–** | **–** | **–** | ● |
| *N. sphaerocarpa* | | UTEX B-2092 | AJ781151 | CAN | **-** | **-** | **-** | **-** | **-** | **–** | **–** | **–** | **–** | **–** |
| *N. sphaerocarpa* | | PCC 7804 | AJ133181 | FRA | **-** | **-** | **-** | **-** | **-** | ● | **–** | **–** | **–** | **–** |
| *N. sphaerocarpa* | | UTEX-B-2093 | AJ781148 | USA | **-** | **-** | **-** | **-** | **-** | **–** | **–** | **–** | **–** | **–** |
| *N. harveyana* | | Hübel1983/300 | DQ185231 | Baltic | **-** | **-** | **-** | **-** | **-** | **–** | ● | **–** | ●^d^ | **–** |
| *N. harveyana* | | Becid27 | AJ781145 | Baltic | **-** | **-** | **-** | **-** | **-** | **–** | **–** | **–** | **–** | **–** |
| *N. harveyana* | | Becid29 | AJ781146 | Baltic | **-** | **-** | **-** | **-** | **-** | **–** | **–** | **–** | **–** | **–** |
| *N. harveyana* | | Bo53 | AJ781143 | Baltic | **-** | **-** | **-** | **-** | **-** | **–** | **–** | **–** | **–** | **–** |
|  | |  |  |  |  |  |  |  |  |  |  |  |  |  |
|  | ^a^Taxonomic scheme as presented previously [12], [13].  ^b^Glycosylated aeruginosin  ^c^deletion removing the *aerI* gene.  ^d^Reported earlier without nodulapeptins [13]. | | | | | | | | | | | | | |

**Table S4** Oligonucleotide primer pairs used to screen 16 strains of *N. spumigena*, 8 strains of *N. sphaerocarpa*, 4 strains *N. harveayana* for the presence of the *aer* gene cluster.

| Primer | Target | Primer sequence, direction 5' → 3' | Length (bp) | Tm (⁰C) | GC (%) |
| --- | --- | --- | --- | --- | --- |
|  |  |  |  |  |  |
| aep1F | *aerM* | GAAGCATTACAACCACAGCG | 20 | 50 | 52 |
| aep1R | *aerM* | GGTTTAACTGCGCTAACTGGTG | 22 | 50 | 55 |
|  |  |  |  |  |  |
| aep2F | *aerB* | GAAGTCAGACCAGATAGCTCCG | 22 | 55 | 57 |
| aep2R | *aerB* | GCGTGGTGCAGTAAAGTTGTCTG | 23 | 52 | 57 |
|  |  |  |  |  |  |
| aep6F | *aerG* | GTGTGCATCATATTCTGGCTG | 21 | 48 | 52 |
| aep6R | *aerG* | GCGCGATCGCTCAATTCCTG | 20 | 60 | 56 |
|  |  |  |  |  |  |
| aep7F | *aerI* | GCTGAACCTCCCAAGATTGG | 20 | 55 | 54 |
| aep7R | *aerI* | GGGTAACTCCACAGACATAG | 20 | 50 | 52 |
|  |  |  |  |  |  |
| aep68F | *aerI* | GCAACTGTACTGGGAGAATTAG | 22 | 45 | 53 |
| aep68R | *aerI* | GGCTAATCCTTGGGCGATCG | 20 | 60 | 56 |
|  |  |  |  |  |  |

**References**

1. Foettinger A, Leitner A, Lindner W (2006) Derivatisation of arginine residues with malondialdehyde for the analysis of peptides and protein digests by LC-ESI-MS/MS. *J Mass Spectro*m 41:623–32.

2. Valls N, López-Canet M, Vallribera M, Bonjoch J (2001) First total syntheses of aeruginosin 298-A and aeruginosin 298-B, based on a stereocontrolled route to the new amino acid 6-hydroxyoctahydroindole-2-carboxylic acid. *Chem Eur J* 7:3446-60.

3. Murakami H, Shin J, Matsuda H, Ishida K, Yamaguchi K (1997) A cyclic peptide, anabaenopeptin B, from the cyanobacterium *Oscillatoria agardhii*. *Phytochemistry* 44:449–452.

4. Banker R, Carmeli S (1999) Inhibitors of serine proteases from a waterbloom of the cyanobacterium *Microcystis* sp. *Tetrahedron* 55:10835-44.

5. Nogle LM, Williamson RT, Gerwick WH (2001) Somamides A and B, two new depsipeptide analogues of dolastatin 13 from a Fijian cyanobacterial assemblage of *Lyngbya majuscula* and *Schizothrix* species. *J Nat Prod* 64:716-19.

6. Kaya K et al. (2002) Spiroidesin, a novel lipopeptide from the cyanobacterium *Anabaena spiroides* that inhibits cell growth of the cyanobacterium *Microcystis aeruginosa*. *J Nat Prod* 65:920-21.

7. Lifshits M, Carmeli S (2012) Metabolites of *Microcystis aeruginosa* bloom material from Lake Kinneret, Israel. *J Nat Prod* 75:209-19.

8. Ishida K et al. (2007) Biosynthesis and structure of aeruginoside 126A and 126B, cyanobacterial peptide glycosides bearing a 2-carboxy-6-hydroxyoctahydroindole moiety. *Chem Biol* 14:565-76.

9. Murakami M, Okita Y, Matsuda H, Okino T, Yamaguchi K (1994) Aeruginosin 298-A, a thrombin and trypsin inhibitor from the blue-green alga *Microcystis aeruginosa* (NIES-98). *Tetrahedron Lett* 36:2785-88.

10. Valls N, López-Canet M, Vallribera M, Bonjoch J (2000) Total synthesis and reassignment of configuration of aeruginosin 298-A. *J Am Chem Soc* 122:11248-49.

11.

Lehtimäki J, Lyra C, Suomalainen S, Sundman P, Rouhiainen L, Paulin L, Salkinoja-Salonen M, Sivonen K. 2000. Characterization of Nodularia strains, cyanobacteria from brackish waters, by genotypic and phenotypic methods. Int J Syst Evol Microbiol. 3:1043-53.

12. Lyra C et al. (2005) Benthic cyanobacteria of the genus *Nodularia* are non-toxic, without gas vacuoles, able to glide and genetically more diverse than planktonic *Nodularia*. *Int J Syst Evol* *Microbiol* 55:555-68.

13. Fewer DP et al. (2009) The nonribosomal assembly and frequent occurrence of the protease inhibitor spumigin in the bloom-forming cyanobacterium *Nodularia spumigena*. *Mol Microbiol* 73:924-37.
